# Genomic Clues of a Multidrug-Resistant Bacterium from Cultured Domestic Silkworm (Bombyx mori L.)

**DOI:** 10.1128/mra.00081-22

**Published:** 2022-05-26

**Authors:** Amit Kumar Mandal, Biraj Sarkar, Hrisikesh Mandal, Arka Pratim Chakraborty, Pradeep Kumar Das Mohapatra, Paulami Dam, Rittick Mondal, Sudip Some, Abdul Sadat, Amit Ghati, Kartik Neog, Sukhendu Mandal, İkbal Agah İnce

**Affiliations:** a Chemical Biology Laboratory, Department of Sericulture, Raiganj University, North Dinajpur, West Bengal, India; b Laboratory of Molecular Bacteriology, Department of Microbiology, University of Calcutta, Kolkata, India; c Mycology and Molecular Plant Pathology, Department of Botany, University of North Bengal, Darjeeling, West Bengal, India; d Department of Botany, Raiganj University, North Dinajpur, West Bengal, India; e Department of Microbiology, Raiganj University, North Dinajpur, West Bengal, India; f Insect Ecology and Conservation Biology Laboratory, Department of Sericulture, Raiganj University, North Dinajpur, West Bengal, India; g Department of Microbiology, Barrackpore Rastraguru Surendranath College, Kolkata, India; h Research Extension Center, Central Silk Board, Ministry of Textiles, Government of India, Samaguri, Dimapur, Nagaland, India; i Department of Medical Microbiology, School of Medicine, Acıbadem Mehmet Ali Aydınlar University, Ataşehir, İstanbul, Turkey; Loyola University Chicago

## Abstract

Enterobacter sp. strain ASE was isolated from the gut of an infected domestic silkworm (Bombyx mori L.; Lepidoptera: Bombycidae). The whole-genome sequence (WGS) of the multidrug-resistant strain Enterobacter sp. ASE, which may contribute to our understanding of the strain’s antibiotic resistance mechanism and virulence properties.

## ANNOUNCEMENT

The gut microbiome plays a pivotal role in the growth and development of silkworms (1). Bombyx mori individuals get infected when they feed on contaminated mulberry leaves (1, 2). Flacherie is a common silkworm disease caused by numerous pathogenic bacteria from diverse genera, like *Aeromonas*, *Bacillus*, Enterobacter, Pseudomonas, *Serratia*, Streptococcus, and Staphylococcus (1, 3, 4). Enterobacter sp. strain ASE was isolated from the gut of a domestic silkworm in our culture facility (1). Antibiotics are commonly used in sericulture businesses to keep silkworms healthy and disease free and enhance cocoon and raw silk production, thus opening the Pandora’s box of antibiotics misuse/overuse (5). Strain ASE is a multidrug-resistant pathogen that has been found to be resistant to a variety of antibiotics, including cell wall inhibitors (carbenicillin, penicillin, bacitracin, mezlocillin, and cefpodoxime), nucleic acid synthesis inhibitors (novobiocin and nitrofurantoin), and protein synthesis inhibitors (oleandomycin and lincomycin) (1). The 16S rRNA gene of isolate ASE (GenBank accession number MT023436) showed 99.65% similarity with Enterobacter hormaechei subsp. *hormaechei* ATCC 49162 (1).

Isolate ASE was stored at −70°C in 20% glycerol and cultured in Luria-Bertani broth (HiMedia; M1245) at 37°C for 18 h for active culturing purposes. Genomic DNA was extracted using the phenol-chloroform method (1, 6). Paired-end libraries were prepared using the NEBNext Ultra DNA library prep kit and sequenced using the Illumina NovaSeq 6000 platform (AgriGenome Labs Pvt Ltd., Kochi, Kerala, India), generating a total of 35,878,887 reads (2 × 151-bp paired-end format) for each direction.

The fastq files were preprocessed using the Adapter Removal version 2.3.1 tool (https://github.com/MikkelSchubert/adapterremoval) to trim all paired-end reads with an average quality score of <30 before performing the assembly (7). *De novo* assembly was performed using the Unicycler version 0.4.8 assembler (https://github.com/rrwick/Unicycler) (8). The assembly produced a draft genome sequence encompassing 56 contigs. The *N*_50_ length is 551,344 bp, while the *L*_50_ count is 4. The estimated genome size is 4,821,076 bp with 55.2% G+C content and 96.46× coverage. The NCBI Prokaryotic Genome Annotation Pipeline (PGAP) version 5.3 was used for annotation with the “best-placed reference protein set” and “GeneMarkS-2+” methods (9). A total of 4,606 coding sequences were annotated, comprising 9 rRNA genes (3 each of 5S, 16S, and 23S) and 75 tRNAs. Further insight into the genome of Enterobacter sp. ASE will undoubtedly help us understand the development of antibiotic resistance in silkworm microbiota.

### Data availability.

This whole-genome shotgun project was deposited at NCBI under the GenBank accession number JAJTNE000000000. The version described in this paper is the first version, JAJTNE010000000, and consists of sequences JAJTNE010000001 to JAJTNE010000056. The BioProject and BioSample accession numbers are PRJNA786204 and SAMN23636983, respectively. The raw data are available at the Sequence Read Archive (SRA) under the accession number SRR17327056.
